# Postural transitions detection and characterization in healthy and patient populations using a single waist sensor

**DOI:** 10.1186/s12984-020-00692-4

**Published:** 2020-06-03

**Authors:** Arash Atrsaei, Farzin Dadashi, Clint Hansen, Elke Warmerdam, Benoît Mariani, Walter Maetzler, Kamiar Aminian

**Affiliations:** 1 Laboratory of Movement Analysis and Measurement, École Polytechnique Fédérale de Lausanne (EPFL), Station 9, Lausanne, 1015 Switzerland; 2 Gait Up SA, EPFL Innovation Park, Bâtiment C, Lausanne, 1015 Switzerland; 3 Department of Neurology, Universitätsklinikum Schleswig-Holstein, Arnold-Heller-Straße 3, Haus 41, Kiel, 24105 Germany

**Keywords:** Postural transition, Sit-to-stand, Activity monitoring, Inertial sensors, Functional test, Mobility, Balance

## Abstract

**Background:**

Sit-to-stand and stand-to-sit transitions are frequent daily functional tasks indicative of muscle power and balance performance. Monitoring these postural transitions with inertial sensors provides an objective tool to assess mobility in both the laboratory and home environment. While the measurement depends on the sensor location, the clinical and everyday use requires high compliance and subject adherence. The objective of this study was to propose a sit-to-stand and stand-to-sit transition detection algorithm that works independently of the sensor location.

**Methods:**

For a location-independent algorithm, the vertical acceleration of the lower back in the global frame was used to detect the postural transitions in daily activities. The detection performance of the algorithm was validated against video observations. To investigate the effect of the location on the kinematic parameters, these parameters were extracted during a five-time sit-to-stand test and were compared for different locations of the sensor on the trunk and lower back.

**Results:**

The proposed detection method demonstrates high accuracy in different populations with a mean positive predictive value (and mean sensitivity) of 98% (95%) for healthy individuals and 89% (89%) for participants with diseases.

**Conclusions:**

The sensor location around the waist did not affect the performance of the algorithm in detecting the sit-to-stand and stand-to-sit transitions. However, regarding the accuracy of the kinematic parameters, the sensors located on the sternum and L5 vertebrae demonstrated the highest reliability.

## Background

Being able to maintain balance during movements is a prerequisite for an independent life. The inability to do so can lead to an increased risk of falls and consequently a dependent and inactive life [1–3]. Balance disorders can lead to problems with postural transitions (PTs), such as the sit-to-stand movements [4]. These challenging PTs require complicated coordination of lower and upper limbs [5] and frequently occur during daily living activities [6, 7]. As the sit-to-stand transitions are indicative of lower limb muscle strength and balance control [6, 8, 9], quantifying these movements is key to understand the underlying problem of balance disorders.

Clinicians conventionally assess the sit-to-stand transitions by either diaries [10] and questionnaires [11, 12] or functional tests. Standardized assessment tools can provide here valuable additional information.

The five-time sit-to-stand (5xSTS) test which measures the time to perform five sit-to-stand transitions [13, 14] and thirty-second chair-rise (30SCT) test which includes the numbers of sit-to-stands that can be performed within thirty seconds [8, 15] are standardized functional tests used in clinical routine to assess the ability to perform, and the quality of transitions. Although these methods have been proven to display discriminative properties for balance disorders [16], subtle differences that may provide further relevant information about the movement are not detectable with these tests [17].

For instance, during sit-to-stand transitions, maximum angular velocity has been shown to be associated with inadequate momentum generation and consequently, the success of the PT [18, 19]. Moreover, duration of each phase of sit-to-stand transitions changes between young and old adults [20] and between older adults with a low or a high risk of fall [21]. Peak power of transition has been reported to be associated with muscle power and strength [22, 23]. Therefore, instrumenting these functional tests and extracting meaningful parameters can provide a more in-depth and precise analysis. Sit-to-stand transitions have been studied with optical motion trackers [24] and force plates [25]. Although these methods provide very detailed and granular information about the movements, they are limited to the laboratory environment [7, 17].

The laboratory setting can only assess the performance of the participants in the confined environment (e.g. in-clinic) while individuals demonstrate different behavior in real-life daily activities [26, 27]. For example, sit-to-stand duration has been shown to be higher during daily activities compared to the functional test performed in the clinic in older adults and in patients with idiopathic Parkinson’s disease (IPS) [28]. Thus, it is important to develop methods that can also be used in domestic environments.

Inertial sensors can be applied in almost every environment. Moreover, they have been already used to instrument the 5xSTS [29] or the 30SCT [30] tests. Kinematic parameters extracted from such instrumented assessments have been shown to have greater clinical relevance than the conventional clinical approach [31, 32]. Wearable sensors have provided an objective tool to evaluate PTs during daily activities as well. Barometric pressure sensor within the pendant device was used as a complementary source of data to detect the PTs [3, 33]; however, due to the pressure changes in outdoor environments, the use of the barometric sensor can adversely affect the detection accuracy. For instance, in [3], the sensitivity of the sit-to-stand detection was decreased by 25% in outdoor environments.

There are some studies on monitoring sit-to-stand transitions with a single inertial sensor on either the sternum or on the lower back. In reference [34], the gyroscope and accelerometer signal along with a discrete wavelet transform have been used to obtain the trunk angle and consequently to detect the PTs. A simpler sensor setup with only a tri-axial accelerometer was used in [35]. In this study, the tilt angle of the trunk was estimated by the scalar product of the accelerometer data and gravity vector obtained during a static calibration at the beginning of each measurement. These studies were validated under very controlled conditions that involved sit-to-stand and stand-to-sit movements with a few other activities. More daily activities were included in the measurement protocol used by [36] and to reduce the false positive trunk movements, fuzzy rules have been employed to improve the accuracy of detection based on the previous or next activity. The performance of the PT detection was further improved by employing a template matching technique with dynamic time warping method in [37]. However, the performance of the detection algorithm was still unsatisfying with a positive predictive value and sensitivity of 22% and 50%, respectively. In another study, with a single inertial sensor on the waist, the candidates of the PTs were first detected by detecting the peaks of the tilt angle of the lower back. These were filtered out by double integrating the vertical acceleration and calculating the elevation change of the lower back [38].

The drawback of all of these studies [21, 34–40] is that they require the sensor to be attached to a specific and fixed location of the body, which is difficult to maintain during daily activities and may not be achievable by patients themselves without a trained operator, thus limiting its broaden applicability in clinical setting.

This issue has been partially solved through using the signal vector magnitude which is the Euclidean norm of the accelerometer signal [41, 42]. The choice of various wavelets and scale approximations were studied in [42] to detect the PTs in a large group of healthy younger and older adults. However, in both of these studies, no method was suggested to distinguish true PTs from movements that can have similar wavelets to PTs. Their algorithms have been validated in measurements involving only sit-to-stand and stand-to-sit movements with rest periods in-between.

To this end, an algorithm which is robust to sensor placement changes and validated in a range of daily activities is desirable. Furthermore, little is known about the transferability of algorithms developed within a certain cohort, to other cohorts (e.g., with different and without diseases). The goal of this study was therefore to evaluate the performance of a new PT detection algorithm in healthy individuals and patients with different diseases that were all equipped with an inertial sensor on different locations around the waist and on the trunk. The new algorithm was validated in both laboratory and daily activity settings. Finally, the effect of the sensors location on the detection performance and extracted parameters was evaluated.

## Methods

### Materials and measurement protocol

In this study, two datasets were used to reach the objectives of the study (Table 1):

- Dataset A: (1) To validate the proposed PT detection method during simple daily activities with inertial sensors on different locations around the waist and on the trunk, (2) to validate the extracted kinematic parameters against reference systems, and (3) to investigate the effect of sensor location on the kinematic parameters

**Table 1:** Demographic data of Datasets A and B

	Population	Participants (female)	Age	Height (cm)	Weight (kg)	Disease scale
Dataset A	Healthy younger adults	15 (4)	27 ±3	172 ±8	67 ±14	-
Dataset B	Healthy younger adults	21 (9)	29 ±9	182 ±8	74 ±12	-
	Healthy older adults	3 (1)	69 ±4	178 ±8	69 ±13	-
	IPS patients	5 (1)	58 ±9	176 ±6	87 ±13	UPDRS^1^: 22 ±7
	MS patients	5 (2)	41 ±17	185 ±5	73 ±8	EDSS^2^: 3 ±2
	Stroke patients	8 (2)	66 ±13	176 ±12	79 ±25	-

^1^Unified Parkinson Disease Rating Scale [43]

^2^Expanded Disability Status Scale [44]
- Dataset B: To demonstrate the performance of PT detection algorithm in different healthy and patient populations

**Fig. 1:**
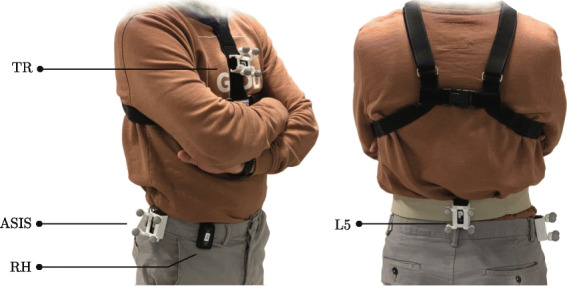
The location of inertial sensors for Dataset A

Dataset A was obtained through measurements on 15 young healthy adults. Table 1 provides demographic information. Participants wore four inertial sensors (Physilog 5, Gait Up, CH) at four different locations on the body (Fig. 1): chest (TR), lower back at the area of L5 (L5), anterior superior iliac spine (ASIS), and an arbitrary position on the right hip (RH). Data from the 3D accelerometer and 3D gyroscope was recorded with a sampling frequency of 128 Hz and was used to test the PT detection algorithm described in the next two sections.

The measurement protocol consisted of two tests. The first test aimed to validate the performance of the PT detection algorithm during 10 minutes recording of daily tasks performed in a fixed order inside a building: sitting on different chairs and sofas with different heights, walking through different offices, bending to pick up objects from the floor, lying, tying shoe laces, picking objects from the fridge, and using stairs and lift. Subjects were free to move outside the lab and between different offices. As the reference events for the PTs, the participants were video recorded during the whole measurement with a camcorder (Sony, Japan) with 25 frames per second.

The goal of the second test was to validate the accuracy of the extracted kinematic parameters and determine the effect of the sensor location on the characterization of the PTs. The participants were asked to perform a 5xSTS test in the lab on a chair without armrest. Regularly, 5xSTS is performed as fast as possible. Here, the test was performed with self-selected speed as this is in our view, closer to daily life behaviour. Two parameters were validated: the trunk tilt angle and the duration of each transition. Trunk tilt was validated by an optical motion capture system (Vicon, UK). Four reflective markers (Fig. 1) were mounted on the inertial sensors to track the movements of the trunk and lower back. Furthermore, the participants were video recorded and transition durations were validated. All subjects were provided with the informed consent, and the protocol was approved by the Human Research Ethics Committee of École Polytechnique Fédérale de Lausanne (EPFL), HREC No: 038- 2018/ 09.08.2018.

Dataset B was obtained through measurements on 42 participants: 21 healthy younger adults, 3 healthy older adults, 5 patients with multiple sclerosis (MS), 5 IPS patients, and 8 patients who had stroke. Table 1 provides clinical and demographic information. The measurement protocol consisted of a home setting simulation in which subjects performed several simple daily living tasks: Setting a table (including sitting at table, eating and drinking, and cleaning table afterwards), standing up and sitting down multiple times (in open space and at a table), ironing, tooth brushing, and replacing objects from different heights and out of a cabinet. As reference for validation, an observer logged the time when the PTs were performed. All participants gave written informed consent and the study was approved by the ethical committee of the medical faculty at Universitätsklinikum Schleswig-Holstein (UKSH), No: D438/18. Since the objective here was to further validate the transition detection algorithm in various populations, data extracted from the L5 sensor (myoMOTION, Noraxon, USA) was used.

### PT detection algorithm

**Fig. 2:**
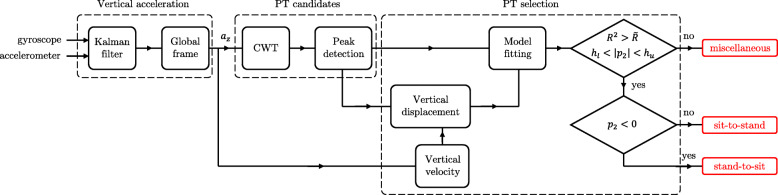
The PT detection algorithm flowchart

The main idea to make the detection algorithm independent of the sensor location was to use the vertical acceleration in the global frame. This vertical acceleration has a positive acceleration peak followed by a negative acceleration peak in the vertical direction during sit-to-stand and a negative peak followed by a positive peak during stand-to-sit transitions [34]. For this purpose, the vertical acceleration in the global frame was obtained first, and then a robust peak detection algorithm was designed to detect the PT candidates. Finally, a fitting model on vertical displacement allowed selecting the actual PTs. The following section describe these different steps. Figure 2, illustrates the algorithm flowchart.

#### Vertical acceleration

Given the measurements from the accelerometer in the sensor frame (*a*_*s*_), the data can be obtained in the global frame by:

1 $$ a_{g} = q \otimes \left[\begin{array}{ll} 0&a_{s} \end{array}\right] \otimes q^{*}   $$

in which *q* is the quaternion specifying the orientation of the sensor in the global frame and is calculated by a Kalman filter fusion of accelerometer and gyroscope (a modified version of the method introduced by [45] in which the measurement model was changed to include only the accelerometer and gyroscope data), *q*^∗^ is the conjugate of the quaternion, the ⊗ operator is quaternion multiplication, and *a*_*g*_ is the accelerometer data in the global frame. Finally, the acceleration of the movement (*a*) can be obtained by subtracting the gravity vector from the accelerometer data in the global frame:

2 $$ a = a_{g} - g   $$

in which $a=\left [\begin {array}{lll} a_{x} &a_{y} &a_{z} \end {array}\right ]$ and *a*_*z*_ is the vertical acceleration. To remove the noise and artifacts included in the signal, a low-pass Butterworth filter of order 12 with a cut-off frequency of 1.3 Hz was used to filter the vertical acceleration. This cut-off frequency was achieved empirically by attenuating other movements than the PTs.

#### PT candidates

As the first step to detect the candidates of these PTs, the continuous wavelet transform (CWT) was applied to detect the specific sit-to-stand and stand-to-sit patterns in *a*_*z*_ [33, 34]. By scaling (frequency localization) and shifting (time localization) of a template signal called mother wavelet, CWT tries to find the patterns through the measured signal similar to the mother wavelet. This will provide us with CWT coefficients (*C*_*w*_(*a*,*t*)):

3 $$ C_{w}(a,t)= \frac{1}{\sqrt{\left | a \right |}}\int_{-\infty }^{+\infty}a_{z}(u){\psi }(\frac{u-t}{a})du  $$

in which, *a* is the scale factor, *t* is the time, *a*_*z*_(*u*) is the vertical acceleration signal, and *ψ*(*u*) is the mother function.

The “bior 1.5" wavelet was chosen as the mother wavelet due to the similarity between this wavelet and the sit-to-stand (or stand-to-sit) vertical acceleration pattern. The coefficients belonging to the scales of 0.5 to 5 seconds (0.2 Hz to 2 Hz) were obtained [33]. The sum of the coefficients was then calculated as:

4 $$ A_{w}(t)= \sum_{a} C_{w}(a,t)  $$

The wavelet analysis was performed by MATLAB Wavelet Analyzer Toolbox. The peaks of the |*A*_*w*_(*t*)| can be chosen as the candidates for the sit-to-stand and stand-to-sit transitions. In order to make the computation more efficient and to avoid less false positives, we have chosen the peaks that are greater than $\frac {1}{4}\text {max}(\left | A_{w}(t)\right |)$, in which max(|*A*_*w*_(*t*)|) is the maximum value of the entire signal of |*A*_*w*_(*t*)|. The reason for using this value rather than a fixed threshold is that individuals have different magnitude of acceleration during PTs. Furthermore, we hypothesized that it is unlikely to have two consecutive PTs within two seconds in the real life settings; thus, the peaks of |*A*_*w*_(*t*)| should have minimum time distance of 2 seconds.

#### PT candidate selection

Since not all the detected candidates belong to the true sit-to-stand and stand-to-sit transitions, it is required to filter out these candidates. For each candidate *k* at time *t*_*k*_, the velocity signal of the movement in the vertical direction (*v*_*z*_) was integrated through an interval of *ΔT* seconds which was set empirically to 4 seconds to get the vertical displacement of the motion throughout the transition:

5 $$ d_{z,k}(t)=\int_{t_{k}-\Delta T/2}^{t_{k}+\Delta T/2} v_{z}(t) dt  $$

where the vertical velocity (*v*_*z*_) was obtained by integrating the acceleration signal throughout the whole measurement and applying a 3rd order Butterworth bandpass filter (0.1–50 Hz) to remove the drift caused by the integration and the noise and bias in the acceleration signal.

**Fig. 3:**
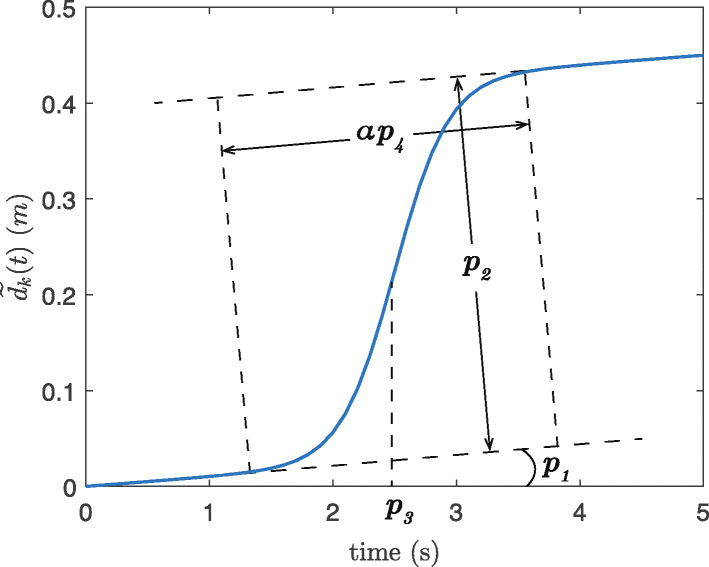
The parameters of the estimated displacement during a sit-to-stand defined by Eq. 6

Upon each *d*_*z*,*k*_(*t*) signal, a Sigmoid model was fitted:

6 $$ \widetilde{d}_{k}(t) = p_{1}t+\frac{p_{2}}{1+\text{exp}{(\frac{p_{3}-t}{p_{4}})}}   $$

in which $\widetilde {d}_{k}(t)$ is the fitted model and *p*_1_, *p*_2_, *p*_3_, and *p*_4_ are the model parameters which were calculated by MATLAB “nlinfit" function. In this model, *p*_1_ accounts for the linear drift, *p*_2_ determines the amplitude of the elevation change, *p*_3_ is the time localization of the PT event, and as it will be explained later, *p*_4_ is linearly proportional to the transition duration, Fig. 3.

A PT candidate *k* is considered as a true sit-to-stand or stand-to-sit if these conditions were satisfied:

- The R-squared (*R*^2^) of the fitting model is above a certain threshold $\widetilde {R}$.
- The elevation change (|*p*_2_|) is between a lower bound *h*_*l*_ and an upper bound *h*_*u*_.

After meeting these requirements, a transition was considered as a sit-to-stand if *p*_2_>0 and a stand-to-sit if *p*_2_<0.

The reason for choosing the *R*^2^ of the fitting model as a metric to detect the true transitions is that this parameter corresponds to the quality of the fitting and specifies the degree of similarity between *d*_*z*,*k*_(*t*) and $\widetilde {d}_{k}(t)$. The value for $\widetilde {R}$ was set empirically to 0.92. The values for *h*_*l*_ and *h*_*u*_ were determined by maximizing the sensitivity and positive predictive value of the detection algorithm based on the L5 location in Dataset A. The same values for the determined parameters were used for other sensor locations in Dataset A and the whole data of Dataset B. A sensitivity analysis was performed to determine the effect of changing the value of these parameters (i.e. $\widetilde {R}$, *h*_*l*_, and *h*_*u*_) on the performance of the detection algorithm.

### Kinematic features

The following kinematic features were extracted to characterize the sit-to-stand and stand-to-sit transitions:

#### Transition duration

Two estimates were used for the transition duration, one based on the angular velocity (*TD*_*ω*_) and the other based on the vertical acceleration (*TD*_*a*_). To calculate *TD*_*ω*_, for each transition, a principal component analysis (PCA) was performed on the gyroscope data, to get the angular velocity of the trunk in the sagittal plane considered as the principal plane for trunk rotation. The start of the transition was defined as the end of the plateau before the negative peak of the angular velocity (forward trunk rotation) and the end of the transition was defined as the start of the plateau after the positive peak of the angular velocity (backward trunk rotation).

For (*TD*_*a*_) estimation, first an approximation of vertical acceleration was obtained by calculating the second derivative of $\widetilde {d}_{k}(t)$:

7 $$ \widetilde{a}_{k}(t)=\frac{d^{2} }{d t^{2} }(\widetilde{d}_{k}(t))   $$

Then using the model presented in Eq. 6 and considering *a*_0_ as the acceleration threshold to define the start and end of plateau, *TD*_*a*_ was obtained by:

8 $$ {TD}_{a}=\alpha p_{4}   $$

in which,

9 $$ \alpha = 2\text{ln}\left(\frac{2\beta}{-2\beta+1-\sqrt{1-4\beta}} \right)   $$

10 $$ \beta=\frac{p_{4}^{2}a_{0}}{p_{2}}   $$

*p*_2_ and *p*_4_ are the displacement model parameters introduced by Eq. 6.

#### Tilt angle and anterior-posterior angular range

The tilt angle (*θ*) was calculated by converting the quaternions to the Euler angles [46].

The anterior-posterior angular range (*Δθ*_*AP*_) was defined as the change in the tilt angle of the trunk at the beginning and the end of the flexion phase (forward trunk rotation) of the sit-to-stand transition.

#### Peak power

The power was calculated by the product of the vertical velocity and the vertical force exerted during the PT [23]:

11 $$ P_{k}(t)=m\widetilde{a}_{k}(t)\widetilde{v}_{k}(t)   $$

in which *m* is the body mass, $\widetilde {v}_{k}(t)=\frac {d }{d t }(\widetilde {d}_{k}(t))$ is the estimated vertical velocity, and $\widetilde {a}_{k}(t)$ is calculated by Eq. 7. The peak power was defined by the maximum power during a transition, i.e. *P*_*max*_=max(*P*_*k*_(*t*))

#### Peak angular velocity

The peak angular velocity (*ω*_*max*_) was defined as the maximum angular velocity during flexion in a sit-to-stand transition in the sagittal plane.

### Validation and statistical analysis

As described before, Dataset A and Dataset B were used for the validation of the PT detection algorithm. The performance of the algorithm was reported by the sensitivity (SE) and positive predictive values (PPV):

12 $$ PPV=\frac{TP}{TP+FP}\times100   $$

13 $$ SE=\frac{TP}{TP+FN}\times100   $$

in which TP stands for true positive, FP for false positive, and FN for false negative.

The second test within Dataset A corresponding to the 5xSTS test was used to estimate the accuracy of the relevant kinematic parameters extracted for the sit-to-stand and stand-to-sit transitions (i.e, *TD*_*ω*_, *TD*_*a*_, *θ*, and *Δθ*_*AP*_) and also to determine the effect of sensor location on the parameters.

For transition duration, two observers logged the durations recorded by the camcorder. The mean of the values determined by the observers was used as the reference. The error was calculated as the difference between the estimated transition duration (*TD*_*ω*_ or *TD*_*a*_) and the reference value. The relative absolute error was also determined.

For the tilt angle, the tilt angles computed by the marker clusters on the TR sensor and L5 sensor were used as the reference. The error was defined as the difference between the reference and the estimated angle by the inertial sensor.

The errors were represented by the mean and standard deviation (std), and the one-sample Kolmogorov-Smirnov test was used to test the normality of the error.

To determine the associations between the parameters obtained by different sensor locations, Pearson’s correlation coefficient (*ρ*) was used. A correlation coefficient of less than 0.5 was considered as low, between 0.5 and 0.7 as moderate, and above 0.7 as high [26].

To show the statistical differences between two measurements, t-test was used where the data is normally distributed; otherwise, Wilcoxon test was employed.

## Results

### Vertical acceleration

**Fig. 4:**
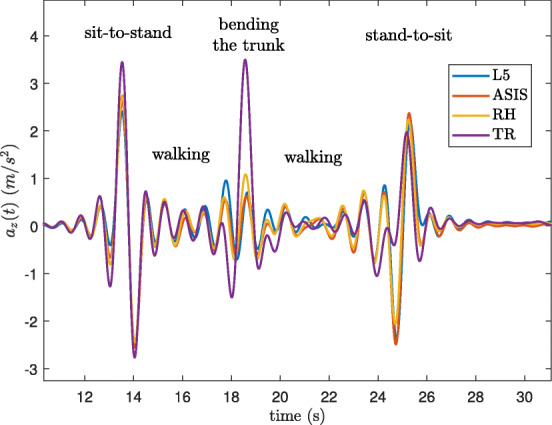
The vertical acceleration signal obtained by different locations of the sensor for a healthy young subject

Figure 4 shows an example of comparing the vertical accelerations (*a*_*z*_) obtained by Eqs. 1 and 2 for data extracted from inertial sensors located at L5, ASIS, RH, and TR, worn by a healthy young participant in Dataset A. The vertical accelerations of the different locations matched almost perfectly. Pearson’s correlation coefficients between respective positions were high, i.e. 0.95 between L5 and ASIS, 0.96 between L5 and RH, 0.94 between L5 and TR, 0.98 between ASIS and RH, 0.91 between ASIS and TR and 0.95 between RH and TR. However, when the participant bent his trunk to pick up an object from the ground, conceivably, higher acceleration in TR was observed compared to L5.

### Vertical displacement

**Fig. 5:**
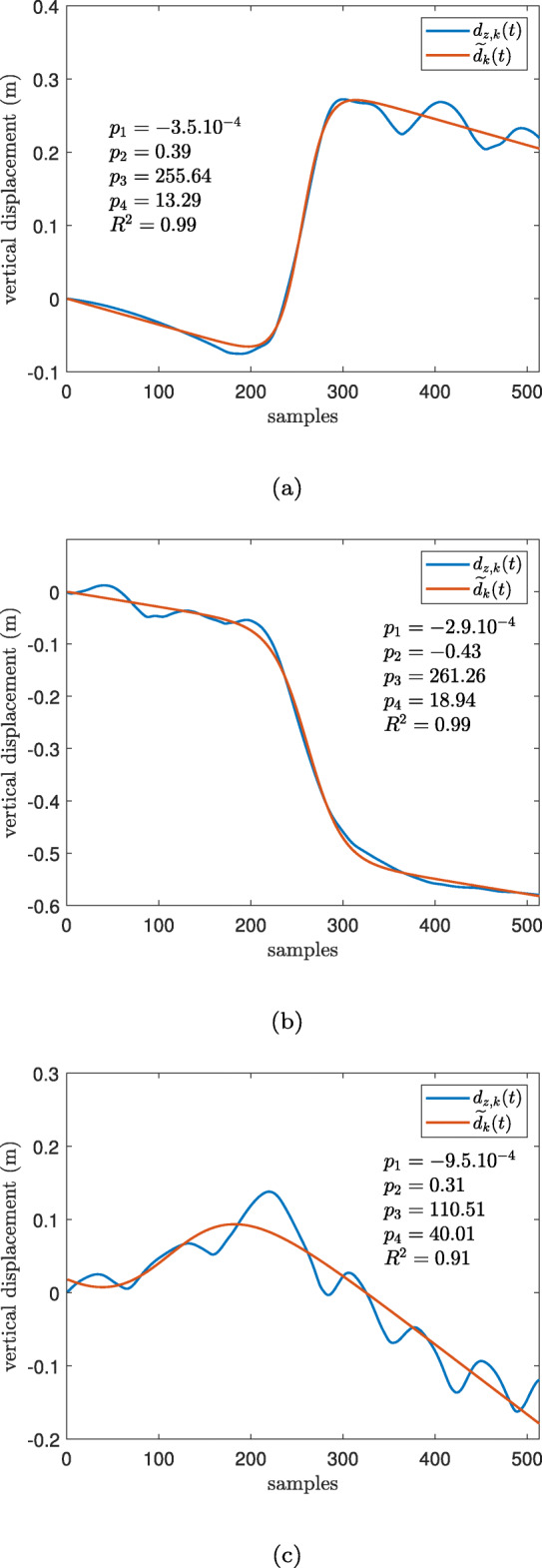
The measured *d*_*z*,*k*_(*t*) (in blue) and estimated $\widetilde {d}_{k}(t)$ (in red) displacement for PT candidates: **a** a sit-to-stand transition **b** a stand-to-sit transition and **c** a miscellaneous movement, *p*_1_ accounts for the linear drift, *p*_2_ determines the amplitude of the elevation change, *p*_3_ is the time localization of the postural transition event, and *p*_4_ is related to the transition duration. *R*^2^ is the R-squared of the fitting

The results of the fitting model for typical sit-to stand and stand-to sit movements were compared with a non-PT transition (miscellaneous movements) and shown on Fig. 5 along with the model parameters.

**Fig. 6:**
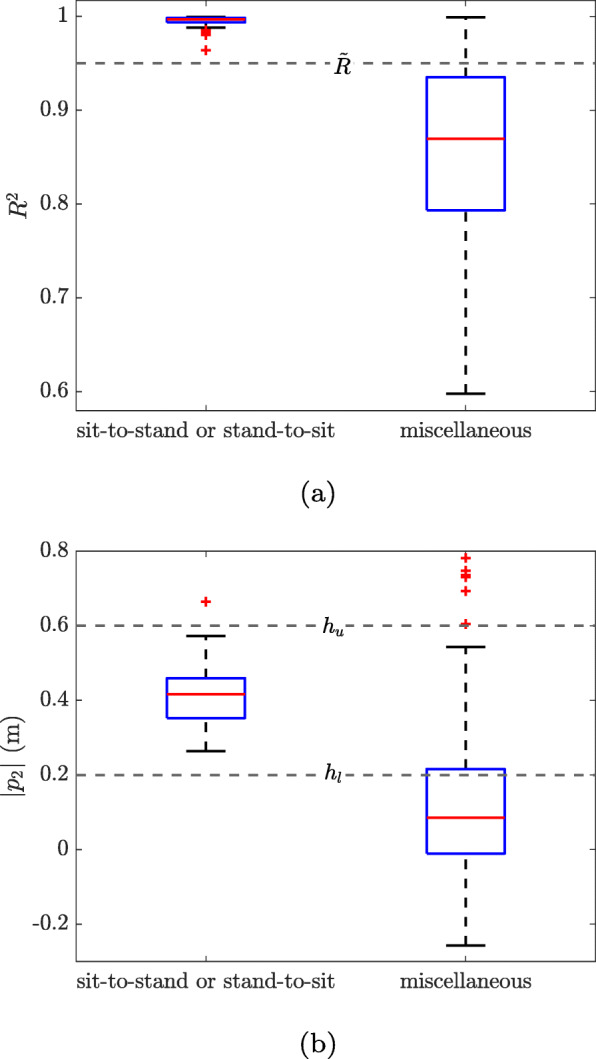
The comparison between the true PTs and miscellaneous movements for **a** the *R*^2^ and **b** the *p*_2_ parameters

For the *R*^2^ and *p*_2_ parameters, the differences between 171 sit-to-stand and stand-to-sit transitions and 35 miscellaneous movements that were detected by the algorithm in Dataset A were shown in Fig. 6. For both of these parameters, the Wilcoxon rank sum test indicated a significant statistical difference between the true transitions and miscellaneous movements (*p*<0.001).

### PT detection

The performance of the algorithm in detecting the sit-to-stand and stand-to-sit transitions during simulated real-life condition were shown in Table 2 for different locations of the sensor (Dataset A) and on Table 3 among different populations for the L5 sensor (Dataset B). Almost the same performance was achieved for all the locations around the lower back, with the ASIS location showing the best performance. The performance was the lowest for TR, which was driven by low sensitivity for stand-to-sit transitions. PPVs for L5, ASIS and RH were above 93%, indicating that only very few miscellaneous movements were detected as PTs by the algorithm.

**Table 2:** Performance metrics defined by Eqs. 12 and 13 for the PT detection algorithm: Dataset A (different sensor location), 15 young healthy adults

	sit-to-stand					stand-to-sit				
	TP	FP	FN	**PPV**	**SE**	TP	FP	FN	**PPV**	**SE**
L5	85	3	5	**97**	**94**	79	6	11	**93**	**88**
ASIS	88	3	2	**97**	**98**	82	6	8	**93**	**91**
RH	81	2	9	**98**	**90**	70	5	20	**93**	**78**
TR	86	14	4	**86**	**96**	68	23	22	**75**	**76**

**Table 3:** Performance metrics defined by Eqs. 12 and 13 for the PT detection algorithm: Dataset B (different population), 21 healthy younger adults, 3 healthy older adults, 5 patients with MS, 5 IPS patients, and 8 stroke patients

	sit-to-stand					stand-to-sit				
	TP	FP	FN	**PPV**	**SE**	TP	FP	FN	**PPV**	**SE**
Healthy younger adults	133	5	8	**96**	**94**	133	0	14	**100**	**90**
Healthy older adults	15	0	0	**100**	**100**	16	1	1	**94**	**94**
IPS patients	24	6	6	**80**	**80**	21	5	9	**81**	**70**
MS patients	23	2	1	**92**	**96**	23	1	4	**96**	**85**
Stroke patients	48	3	4	**97**	**92**	72	5	18	**94**	**80**

As reported in Table 3, the algorithm achieved lower performance among IPS patients while for the other populations the performance was high.

It should be mentioned that the threshold for the R-squared ($\tilde {R}$) was set empirically to 0.92. By the sensitivity analysis, it was observed that a change of ±2*%* in the value of $\tilde {R}$ will affect the PPV and SE values by ±1*%*. Furthermore, the *h*_*l*_ and *h*_*u*_ values determined by maximizing the mean of the PPV and SE of the sit-to-stand and stand-to-sit detections were 20 cm and 60 cm, respectively. A change of ±5 cm for these values affect the PPV and SE parameters by ±1*%*.

### Kinematic features

The algorithm detected all PTs correctly that were performed during the 5xSTS test. The kinematic features defined previously were extracted and compared for different sensor locations; where applicable, the parameters were validated against the reference system.

#### Transition duration

Regarding the difference between the transition durations, the Wilcoxon signed rank test showed no significant difference between the observers for the sit-to-stand transitions (*p*>0.05); however, there was a significant difference for the stand-to-sit transitions (*p*=0.02).

Among the two methods proposed for the estimation of the transition duration, (*TD*_*ω*_) which was based on the angular velocity had lower errors for all of the locations compared to the method based on vertical acceleration (*TD*_*a*_). Overall, for both of the methods, the accuracy of the L5 sensor was the highest, followed by the TR sensor; whereas, RH sensor was the least accurate (Table 4). The relative absolute error for both of the methods for the L5 location was calculated. The 75th percentile of the relative error for *TD*_*ω*_ was 9.8% and 6.6% for the sit-to-stand and stand-to- sit durations, respectively while these values for *TD*_*a*_ were 18.8% and 24.0%.

**Table 4:** Mean (standard deviation) of the error (in milliseconds) of the transition duration (*TD*_*ω*_ and *TD*_*a*_) compared to reference values obtained by the observers

	sit-to-stand				stand-to-sit			
	L5	ASIS	RH	TR	L5	ASIS	RH	TR
*TD*_*ω*_	-2 (233)	338 (297)	229 (310)	-20 (229)	-27 (172)	237 (337)	101 (319)	-43 (165)
*TD*_*a*_	-18 (387)	54 (344)	208 (564)	-21 (318)	224 (275)	160 (352)	312 (539)	80 (281)

#### Tilt angle and anterior-posterior angular range

**Fig. 7:**
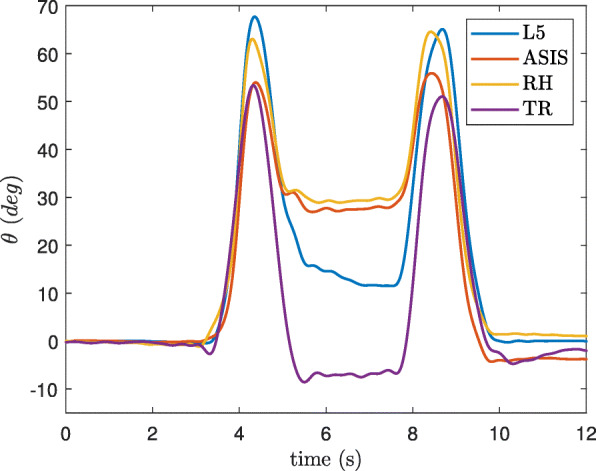
The tilt angle of the trunk obtained by the inertial sensors on different locations during one trial of a sit-to-stand and stand-to-sit

The tilt angle during a typical trial of the sit-to-stand and stand-to-sit transitions is shown on Fig. 7 in which it is observed that the angular range was underestimated by the ASIS and RH sensors.

The error of the total tilt angle signal (*θ*) and the anterior-posterior angular range (*Δθ*_*AP*_) were compared to the references obtained by the optical motion tracker (Table 5). *θ* was obtained during the whole 5xSTS test of all the participants and *Δθ*_*AP*_ was calculated for 5 sit-to-stand transitions giving 75 values for all the participants. The lowest errors among the sensors belonged to L5 and TR locations with the 75th percentile relative absolute error of 5.6% and 8.1%, respectively.

**Table 5:** Mean (standard deviation) of the error of the tilt angle (*θ*) and the anterior-posterior angular range (*Δθ*_*AP*_) compared to the reference system (Ref.) in degrees

Location	L5	ASIS	RH	TR
Ref.	L5			TR
*θ*	-0.3 (2.1)	-6.7 (10.7)	-4.8 (6.9)	-1.6 (3.3)
*Δθ*_*AP*_	-1.0 (3.2)	-6.1 (9.0)	1.2 (11.8)	-1.5 (3.0)

For sensors around the belt, the reference was the L5 sensor

#### Peak power

**Fig. 8:**
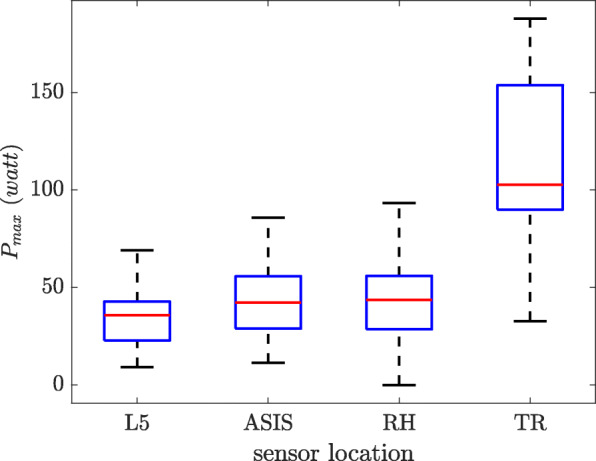
The peak power calculated by different sensor locations

For each subject the peak power was calculated for each of the five sit-to-stand transitions, providing 75 values for each sensor location. The box plot on Fig. 8 shows these values for different sensor locations. The sensors around the belt had almost the same range while trunk sensor shows higher values. High correlations were found between the L5, ASIS, and TR sensors (0.95 between L5 and ASIS, 0.77 between L5 and TR, 0.78 between ASIS and TR). Furthermore, moderate to high correlations were determined between the sensors around the belt (0.65 between L5 and RH and 0.74 between ASIS and RH). The correlation between RH and TR was 0.44.

#### Peak angular velocity

**Fig. 9:**
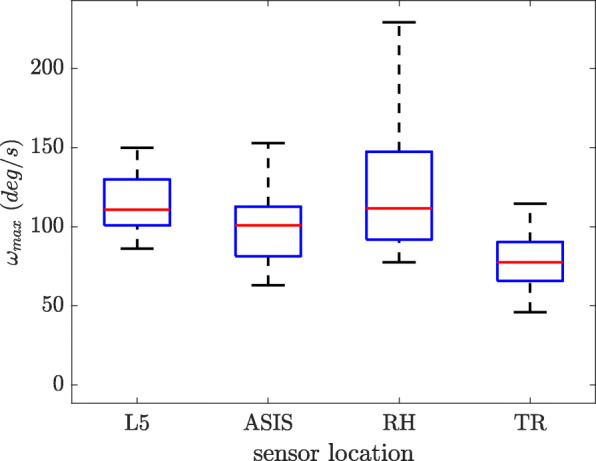
The peak angular velocity calculated by different sensor locations

For each subject the peak angular velocity was calculated for each of the five sit-to-stand transitions, providing 75 values for each sensor location. The box plot for the peak angular velocity calculated by different sensor locations is shown on Fig. 9. The peak angular velocity determined by the trunk sensor was the lowest among all the locations. The correlation coefficient values were obtained as 0.7 between L5 and ASIS, 0.52 between L5 and RH, 0.54 between L5 and TR, 0.67 between ASIS and RH, 0.35 between ASIS and TR and 0.47 between RH and TR.

### Comparison of kinematic parameters between populations

**Fig. 10:**
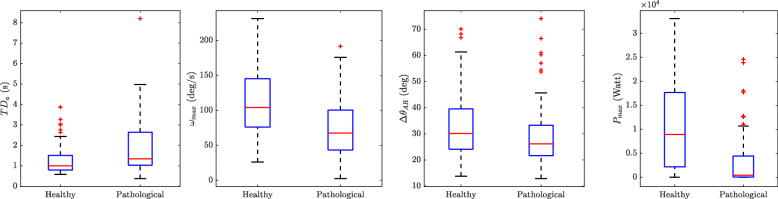
Comparison of the kinematic parameters between the healthy and pathological participants in Dataset B

The extracted kinematic parameters from Dataset B (i.e. *TD*_*a*_, *ω*_*max*_, *Δθ*_*AR*_, and *P*_*max*_) were compared between healthy (24 participants) and pathological (18 participants) groups (Fig. 10). *TD*_*a*_ was significantly lower (*p*<0.05) in healthy subjects compared to the patient population, while *ω*_*max*_, *Δθ*_*AR*_, and *P*_*max*_ were significantly higher (*p*<0.05) for healthy participants. To investigate the effect size, the Cohend’s d for *TD*_*a*_, *ω*_*max*_, *Δθ*_*AR*_, and *P*_*max*_ were obtained as 0.6, 0.8, 0.2, and 0.7 respectively.

## Discussion

The goal of this study was to develop and validate an algorithm to detect sit-to-stand and stand-to-sit transitions in healthy individuals and patients and provide useful parameters for functional evaluation. The algorithm is applicable on a single inertial sensor around the waist or on the trunk making the system appropriate for daily and clinical use. The algorithm validated in the lab showed high performance and its use in the field demonstrated little sensitivity to the change between healthy and pathological group.

Utilizing the vertical acceleration in the global coordinate system made the algorithm robust to sensor placement changes. The estimated vertical acceleration in the global frame was actually similar between different locations around the trunk based on high correlations observed between signals that were extracted from different sensors. Therefore, our hypothesis regarding the similarity between the vertical accelerations produced by different sensor locations seems valid even if some discrepancy can be observed in Fig. 4. Not surprisingly, the ASIS and RH sensor positions which had the closest distance to each other, had the highest correlation values while the sensors at TR and ASIS positions had the lowest correlation, because they were relatively distant from each other.

The performance of the algorithm in detecting the PTs were validated against video observations (Tables 2 and 3). The protocol of the test included a broad range of simple activities of daily living rather than only isolated PTs as used by [21, 34, 35, 39, 41, 42, 47]. The algorithm showed an excellent performance in detecting these transitions with the inertial sensors around the waist (PPV of more than 97% and SE of more than 90%). However the TR sensor, showed lower accuracy. During a PT, the upper back performs more rotation than the lower back area, and because the algorithm was developed based on the lower back displacement model, this aspect may best explain this phenomenon. The differences of rotation values between these body areas were confirmed by calculations with the tilt angle, where the flexion and extension angular ranges were lower in the L5, ASIS and RH positions, than in the TR position (Fig. 7).

The ASIS position was the most accurate in PT detection (Table 2) which probably was due to the rigid attachment of the sensor to this position (Fig. 1). RH and L5 sensors may be exposed to some artificial motion, occurring, e.g., from soft-tissue movement and less stable positioning on the body.

Compared to previous studies [3, 33, 36–38] with almost the same measurement protocol, our algorithm demonstrated better performance in detecting PTs, with a mean PPV and SE of 98% and 95% for healthy adults and 89% and 89% for participants suffering from diverse diseases. With a pendant device used by 25 community-dwelling older people, the performance of the algorithm used in [33] had a SE of 93% and a PPV of 90%. Moreover, compared to this study, we did not use the barometric pressure sensor, as the pressure changes from one place to another might affect the accuracy of the algorithm. In studies [36] and [37] in which a single inertial sensor on chest was used, the SE and PPV assessed through a group of 15 younger adults in a controlled protocol were less than 80% [48]. Compared to a study in which a single inertial sensor on lower back was used [38], our algorithm showed better performance in healthy older adults. In IPS patients without dyskinesias, the former study reached higher PPV and SE than our study. The most probable explanation is that in IPS patients the duration of the PTs may be longer compared to the healthy subjects [28] and our displacement model might not capture the high amount of the drift.

Compared to the study in [40], our algorithm had higher SE in sit-to-stand detection but slightly lower SE in stand-to-sit transitions. The lower SE in detecting stand-to-sit movements by our method might be attributed to the fact that sometimes after sitting down, people try to adjust their posture on the chair and perform one or two smaller PTs right after the original stand-to-sit. Therefore, their vertical displacement does not comply with the sigmoid model presented in Fig. 5b. The reduced accuracy in detecting the stand-to-sit movements has been also observed in [42] in which the authors have considered the various strategies of individuals in sitting down as the contributing factor. Our hypothesis is in agreement with their statement.

In previous works, the inertial sensor was always placed on either TR or L5 [21, 34–38, 42, 47]. We are not aware of any study that investigated PTs using different inertial sensor positions on the human body simultaneously. We investigated the effect of different sensor locations on the kinematic parameters during the PTs during the 5xSTS test.

**Fig. 11:**
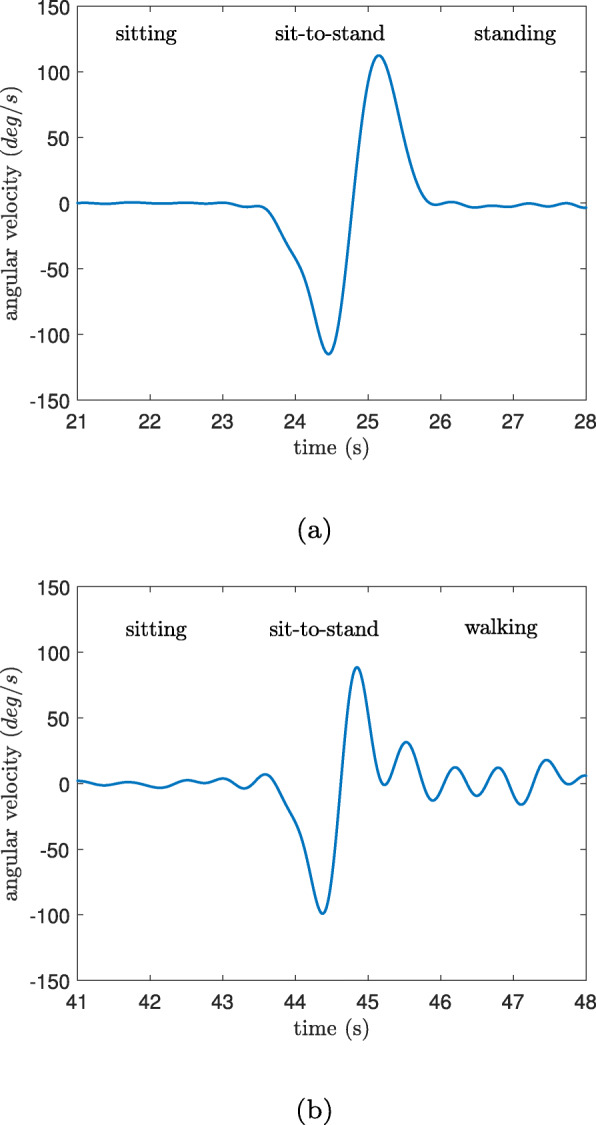
The angular velocity of the trunk for a young healthy subject (a) during the 5xSTS test and (b) real life setting

**Fig. 12:**
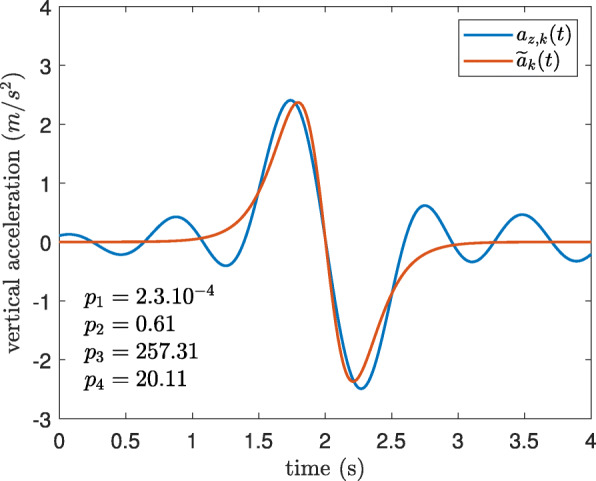
The fitted acceleration model for a sit-to-stand transition during daily activities for a young healthy subject, *a*_*z*,*k*_(*t*) is the measured acceleration and $\widetilde {a}_{k}(t)$ is the estimated acceleration

In order to estimate transition duration, the angular velocity method (*TD*_*ω*_) showed a better accuracy (34% less error); however, the acceleration-based approach (*TD*_*a*_) is preferable in real life situations. Because, during the 5xSTS test, only sit-to-stand and stand-to-sit movements with rest periods in between were measured which allows the detection of the angular velocity plateau (Fig. 11a); however, this is not the case in daily activities as there are additional movements involved, e.g. walking after sit-to-stand movement (Fig. 11b). In fact, the estimated model of the vertical acceleration ($\widetilde {a}_{k}(t)$), isolates the PT movement from the signal (Fig. 12), and with the help of the parameters of the fitted model, the transition duration can be determined by Eqs. 8-10.

Interestingly, although comparable results were obtained for the sit-to-stand phase, there was a statistically significant difference between the two observers concerning the estimation of stand-to-sit phases. Although we do not have any explanation for this observed difference, we see this result as a further argument for the use of objective measurement techniques, as provided by inertial sensors for instance and the algorithm introduced here.

Compared to the previous studies that validated the transition duration against video observations [3, 38, 42, 47], our algorithm achieved higher accuracy with a bias of 2 (L5 location) and 20 (TR location) milliseconds in sit-to-stand and 27 (L5 location) and 43 (TR location) milliseconds for stand-to-sits. The bias of the error was obtained as 10 to 50 milliseconds for sit-to-stand and 80 to 170 milliseconds for stand-to-sits in [42, 47]. In [38], the bias of the error was 200 milliseconds compared to the video observations. Furthermore, the relative absolute error for L5 location was less than 24% as obtained by [3] from a pendant device.

The peak power was overestimated by the chest sensor (Fig. 8) compared to the other three placements, as it is at the proximal distance relative to the lower back; therefore, during a rotation, it undergoes higher vertical velocity and acceleration (Eq. 11). However, in spite of the differences between the upper and the lower back peak power, high correlations were found for all of the locations. Since peak power is based on vertical acceleration and velocity, it can be considered as a metric that is more robust to the changes in sensor location.

Comparing the peak angular velocity for different locations, lower correlations were found for the TR and RH sensors with respect to the other two locations. For the RH sensor as it is hinged to the belt with a rubber clip, the abdominal muscles may push the sensor around the belt, causing artifacts that are not related to the postural movement itself. This also explains the higher range of peak angular velocity calculated by the RH sensor compared to the other locations (Fig. 9). The low correlations with TR sensor, can be explained by different rotational behavior of upper and lower back.

Finally, the comparison between healthy and pathological participants in the extracted kinematic parameters showed that our algorithm was able to show the subtle differences between different populations in an objective manner. The Cohen’s d values for these parameters revealed that for the peak angular velocity and peak power the difference between the healthy and patient populations were greater than the angular range and duration of the transition. Yet further studies with bigger sample group are needed to investigate in details the association of those parameters with specific disease symptoms.

One limitation of our study is the use of wavelet transform as it is computationally expensive and may not be appropriate for real-time applications. To compare the signal to the PT templates, cross correlation can be used instead of the wavelet transform. Moreover, the use of only accelerometer data rather than the fusion of accelerometer and gyroscope data should be studied in order to decrease the power consumption of the device [35].

As there was a variety of populations performing the PTs in this study, the discriminative power of the kinematic parameters could be studied. It has been shown in [32] that the spatiotemporal and kinematic parameters extracted during sit-to-stand transitions can help clinicians detect individuals with frailty and abnormal functional capacities.

## Conclusion

This study presents a novel algorithm for detecting the sit-to-stand and stand-to-sit transitions in both the simulated home setting and the laboratory environment based on a single inertial sensor. The novelty of this approach is that the algorithm is largely independent of the position of the inertial sensor on the trunk. The algorithm was validated in both healthy subjects and patients suffering from diverse diseases in simulated daily activity situations. This study used a novel approach to estimate the transition duration and peak power, by introducing a fitting model on the vertical displacement of the trunk. The effect of the location of the sensor on the extracted kinematic parameters was also investigated, and it was shown that the L5 and TR positions are the most accurate locations to evaluate transition duration and tilt angle of the PTs.

Further research should now investigate the predictive and discriminative power of the kinematic parameters from the novel PT detection algorithm, for different aging and diseased populations.

## Data Availability

The datasets generated and/or analyzed during the current study are not publicly available (no ethical committee approval) but are available from the authors on reasonable request.

## Abbreviations

3D: Three dimensional
30CTS: Thirty-second chair rise
5xSTS: Five-time sit-to-stand
ASIS: Anterior superior iliac spine
CWT: Continuous wavelet transform
FN: False negative
FP: False positive
IPS: Idiopathic Parkinson’s disease
MS: Multiple sclerosis
PCA: Principal component analysis
PPV: Positive predictive value
PT: Postural transition
RAE: Relative absolute error
TP: True positive
TR: Trunk
SE: Sensitivity
std: Standard deviation
